# Overnight Immune Regulation and Subjective Measures of Sleep: A Three Night Observational Study in Adolescent Track and Field Athletes

**DOI:** 10.3389/fspor.2021.689805

**Published:** 2021-09-28

**Authors:** Thomas Steidten, Philipp Baumbach, Rico May, Brunhild Gabriel, Marco Herbsleb, Adrian Markov, Urs Granacher, Michael Kellmann, Wilhelm Bloch, Holger H. W. Gabriel, Christian Puta

**Affiliations:** ^1^Department of Sports Medicine and Health Promotion, Friedrich-Schiller-University Jena, Jena, Germany; ^2^Center for Interdisciplinary Prevention of Diseases Related to Professional Activities, Friedrich Schiller University, Jena, Germany; ^3^Department of Anesthesiology and Intensive Care Medicine, University Hospital Jena, Jena, Germany; ^4^Physical Education/English/Sports Theory, Sports High School, Johann Chr. Fr. GutsMuths Jena, Jena, Germany; ^5^Division of Training and Movement Sciences, Research Focus Cognition Sciences, University of Potsdam, Potsdam, Germany; ^6^Faculty of Sport Science, Ruhr University, Bochum, Germany; ^7^School of Human Movement and Nutrition Sciences, The University of Queensland, St. Lucia, QLD, Australia; ^8^Department of Molecular and Cellular Sport Medicine, German Sport University Cologne, Cologne, Germany

**Keywords:** leukocytes, sleep, adolescents, immune regulation, athletes, track and field training

## Abstract

To ensure health maintenance of young athletes, immunological stress due to physical exercise has to be balanced for performance development and health maintenance. Sleep is an important influencing factor for immune regulation because of its regenerating effect. In an attempt to assess overnight immune regulation, this observational study aimed to examine associations between changes in capillary immunological blood markers and measures of sleep in adolescent athletes. Over a period of three nights, 12 male (*n* = 6) and female (*n* = 6) adolescent track and field athletes aged 16.4 ± 1.1 years were monitored for their sleep behavior (e.g., sleep duration, sleep depth) and immune regulation by using subjective (e.g., sleep) and objective (capillary blood markers) measurement tools. Over the 4 day (three nights), athletes followed their daily routines (school, homework, free time activities, and training). Training was performed for different disciplines (sprint, hurdles, and long-jump) following their daily training routines. Training included dynamic core stability training, coordination training, speed training, resistance training, and endurance training. Capillary blood samples were taken 30–45 min after the last training session (10:00–12:00 a.m. or 5:00–6:00 p.m.) and every morning between 7:00 and 10:00 a.m. Changes in capillary blood markers from post-training to the next morning and morning-to-morning fluctuations in capillary blood markers were analyzed over a three-night period using a generalized estimating equations (GEE) statistical approach. Associations of overnight changes with measures of sleep were analyzed using GEE. We found significant decreases in white blood cell count (WBC), granulocytes (GRAN), granulocytes% (GRAN%), monocytes (MID), and granulocyte-lymphocyte-ratio. In contrast, lymphocytes% (LYM%) increased significantly and systemic inflammation index showed no difference from post-training to the next morning. Furthermore, there was a significant decrease in WBC and GRAN between morning 1 and morning 3. At morning 4, values returned to baseline (morning 1), irrespective if athletes performed a training session or rested on day 3. Furthermore, sleep duration was significantly and negatively associated with changes in WBC (β*z* = −0.491) and lymphocytes (β*z* = −0.451). Our results indicate that overnight sleep duration is an important parameter of immunological overnight regulation for adolescent athletes.

## Introduction

Immunological regulation due to physical exercise and recovery during sleep has to be considered for performance development and health maintenance in athletes. Insufficient sleep over night (<7 h/night) and poor sleep quality are very important features for the immunological recovery and performance of athletes (Walsh et al., 2021). Therefore, it seems timely to examine physiological train due to exercise and recovery by taking the relationship of sleep and immune function into account.

There is evidence in the literature (Gleeson et al., 2000; Moreira et al., 2013; Freitas et al., 2016; Moraes et al., 2017; Puta et al., 2018), that intense resistance and endurance training induces acute immunological stress responses in adolescent athletes. Accordingly, Puta et al. (2018) found significant increases in white blood cells (WBC), granulocytes (GRAN) and granulocytes% (GRAN%) from morning to post-training sessions. Furthermore, the authors reported a significant decrease in lymphocytes% (LYM%) but not lymphocytes (LYM), which was most probably related to the increase in GRAN. Given that physical exercise induces acute changes in immune function, cell concentration and activity (Gabriel and Kindermann, 1997; Pedersen, 2000; Campbell and Turner, 2018; Puta et al., 2018), the risk of infection may even be higher in adolescent athletes (Nieman and Wentz, 2019).

Sleep is an important candidate for immune regulation (Irwin and Opp, 2017; Besedovsky et al., 2019; Prather, 2019). The link between sleep and immunity is based on the interaction with the autonomic nervous system (ANS) and the hypothalamo pituitary adrenal (HPA) axis (Prather, 2019). During overnight sleep, the sympathetic nervous system (SNS) and HPA axis are downregulated (Lange et al., 2010; Irwin and Opp, 2017) resulting in lower catecholamine release (Born et al., 1997; Lange et al., 2010; Del Gallo et al., 2014; Mohamed and Özer, 2019). This process affects immune cells (Prather, 2019) and cytokines (Del Gallo et al., 2014; Ibarra-Coronado et al., 2015; Irwin and Opp, 2017), leading to reduced circulating counts of neutrophil granulocytes (NEUT) in the morning. In contrast, LYM concentrations are increased compared to evening levels (Born et al., 1997; Lange and Born, 2011). More specifically, there is evidence that shorter sleep duration is associated with increased WBC and GRAN levels compared to normal sleep duration (Dinges et al., 1994; Born et al., 1997; Kerkhofs et al., 2007; Boudjeltia et al., 2008; Faraut et al., 2011; Pérez de Heredia et al., 2014). For instance, Pérez de Heredia et al. (2014) showed a significantly negative association of sleep duration with WBC, NEUT and monocytes in a cross sectional study including 933 adolescents aged 14.9 ± 1.2 years (range 12.5–17.5 years). In a cross-over study, Born et al. (1997) observed acute changes in immune cells and mediators after both, a single night of normal sleep and a single night of total sleep restriction. Results showed significantly higher WBC and LYM cell counts during restriction, compared to 8 h of sleep. Longitudinal studies examining the impact of sleep duration on immune cells primarily investigate the influence of sleep deprivation (Kerkhofs et al., 2007; Boudjeltia et al., 2008; Faraut et al., 2011) or sleep loss (Dinges et al., 1994; Born et al., 1997). For example, Boudjeltia et al. (2008) investigated the effect of three nights of sleep restriction (4 h sleep per night) in eight young men (24.5 ± 3.3 years). Morning measurements of the controlled trial revealed significant increases in WBC and NEUT after three nights of sleep restriction, while controls showed no significant changes. Abedelmalek et al. (2013) showed that a single night of partial sleep restriction (10:30 p.m. to 3:00 a.m.) affects pro-inflammatory cytokine regulation in response to repeated brief anaerobic exercise intervals. More precisely, interleukin-6 and tumor necrosis factor-alpha concentrations increased 60 min. after exercise compared to a regular sleep duration (10:30 p.m. to 6:00 a.m.). Further, early rising times seem to additionally affect anerobic performance in the afternoon but not in the morning (Souissi et al., 2013). During periods of rapid growth and/or intense training, adolescent athletes have to additionally cope with psycho-social and environmental stressors (Bermon et al., 2017). The sum of the interacting stressors has to be balanced through adequate coping strategies including sufficient recovery and sleep (Bird, 2013; Walsh et al., 2021). Rapid growth during adolescence and concomitant hormonal and neuronal changes that lead to disturbed sleep-wake rhythm affect sleep duration and sleep quality (Campbell et al., 2007; Hagenauer et al., 2009; Darchia and Cervena, 2014). Besides, sleep is negatively affected by general factors (e.g., early school start times; Au et al., 2014) and individual behavior patterns (screen time, caffeine intake, and mental health issues; Falbe et al., 2015; Owens, 2015). Impaired sleep is associated with HPA axis and SNS activation, which induces catecholamine release and pro-inflammatory cytokine production (Abedelmalek et al., 2013; Besedovsky et al., 2019). This in turn, negatively affects immune regulation. Due to impaired sleep, it is likely that immune regulation is insufficient before starting another training session. Furthermore, immunosuppression may accumulate over the weekly training routine. Consequently, it is necessary to evaluate subjective (i.e., sleep) and objective measures (i.e., capillary immunological blood markers) of overnight immune regulation. Thus, it is crucial to evaluate overnight immune regulation in adolescent athletes.

Therefore, the aim of this observational study was to examine the associations of post-training to next morning changes in capillary immunological blood markers (white blood cells, lymphocytes, lymphocytes%, monocytes, monocytes%, granulocytes, granulocytes%, and platelets) and subjective measures of sleep (e.g., sleep duration and sleep depth) in male and female adolescent track and field athletes. In accordance, with Born et al. (1997), Prather (2019), and Irwin and Opp (2017), we hypothesized, that overnight immune regulation is characterized by decreases in leukocytes. With respect to the relevant literature concerning the interaction of sleep and immune regulation (Born et al., 1997; Lange and Born, 2011; Faraut et al., 2012; Ingram et al., 2015; Besedovsky et al., 2019), we expected that overnight regulation in leukocytes and related inflammatory indexes (granulocyte-lymphocyte-ratio, systemic inflammation index) are negatively associated with measures of sleep (e.g., sleep duration and sleep depth).

## Materials and Methods

### Study Design

**Figure 1:**
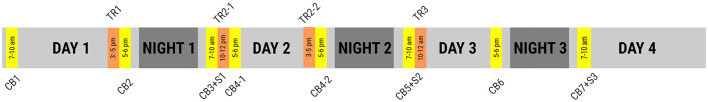
Study protocol of capillary blood measurements (CB), sleep assessment (S), and track and field training sessions (TR).

Over the three night study period, was performed as a repeated measurement design with tests on seven occasions (four morning, three post-training). In order to reflect overall day stress (physical and psychosocial) and its regulation overnight, measurements were taken after the last training session of the day and in the morning. The study ended after the morning measurement on day 4. Capillary immunological blood markers were always taken after the last training session of the day and in the morning, whereas subjective measures were only conducted in the morning (see Figure 1). Infectious causes during the study were identified using a questionnaire on signs of upper and lower respiratory infections. Participants who showed symptoms of upper or lower respiratory infections for longer than 48 h (Spence et al., 2007) were excluded from further analyses.

### Participants

Participant recruitment was applied with reference to the conceptual model of long-term athlete development (Granacher et al., 2016). As our study focused on adolescent athletes only, participants were aged 12–18 years with pubertal Tanner stage classification of III-IV (Granacher et al., 2016). Participants were recruited from the sports high school “Johann Chr. Fr. GutsMuths” Jena and had to be experienced with balance training, plyometric training, core strength training, free weight training and heavy resistance strength training. Accordingly, 14 (seven females and seven males) track and field athletes (age: 16.4 ± 1.1 years, height: 179.9 ± 9.9 cm, weight: 68, 89 ± 1.04 kg) with 6–8 years of experience and a training volume of 10–12 h per week (including 2 h physical education, 4 h sports specific physical education) were eligible for inclusion and recruited to participate in this study. Athletes participated in the disciplines sprint (100, 200, and 400 m), hurdles (female: 100 m; males: 110 m) and long-jump. Prior to the start of the study, athletes had to be free from signs and symptoms of upper and lower respiratory tract infections for at least 2 weeks. In accordance with Spence et al. (2007), athletes suffering from those signs and symptoms for more than 48 h were excluded from further analyses. Written informed consent was obtained from the athletes and their legal representatives prior to the start of the study. Procedures, benefits and risks of the study were explained to the athletes and their legal representatives. This study was carried out in accordance with the recommendations of the University Research Ethics Committee of the Friedrich-Schiller-University Jena, Germany and the latest version of the Declaration of Helsinki. The Protocol was approved by the University Research Ethics Committee of the Friedrich-Schiller-University Jena (458510/15).

### Subjective Sleep Quality

Measures of sleep were obtained by using a questionnaire, which was adopted from the Pittsburgh Sleep Quality Index (PSQI; Buysse et al., 1989) and Richards Campbell Sleep Questionnaire (RCSQ, Richards et al., 2000). Single items of the PSQI were translated into German and used for sleep assessment. Intercorrelation coefficient (ICC) for “Sleep Duration” (ICC = 0.80) has already been reported (Buysse et al., 1989). In addition, “Sleep Depth” was added with reference to the RCSQ.

### Objective Measures

Capillary immunological blood markers were measured using a 20 μl capillary blood sample, taken from the earlobe. The measurements took place at every morning between 8:00 and 10:00 a.m. and 30–45 min after the last training session of the day. White blood cells (WBC), lymphocytes (LYM), lymphocytes% (LYM%), monocytes (MID), monocytes% (MID%), granulocytes (GRAN), granulocytes% (GRAN%), and platelets (PLT) were analyzed using a medonic hematology system (Medonic M16M, Boule Medical AB, Spånga, Sweden). Inflammatory indexes were used in order to assess systemic inflammation sensitivity for immune regulation. The granulocyte-lymphocyte-ratio (GLR) was calculated by using the following formula: $GLR=\;\frac{GRAN}{LYM}$. The systemic inflammation index (SII) was calculated $SII=PLT{\;}^{*}\frac{GRAN}{LYM}$ (Hu et al., 2014). Intra-Assay Coefficients of Variability for Micro Pipette Adapters were recently provided by the manufacturer (WBC ≤ 2.5%, PLT ≤ 3.0%, HGB ≤ 1.3%; Medical AB, Spånga, Sweden). Our own measurements revealed acceptable Intra-Assay Coefficients of Variability for WBC ≤ 2.22%, GRAN ≤ 2.14%, GRAN% ≤ 1.42%, LYM ≤ 2.95%, LYM% ≤ 1.20%. Inter-Assay Coefficients of Variability were also acceptable with WBC ≤ 1.06%, GRAN ≤ 1.40%, GRAN% ≤ 1.39%, LYM ≤ 2.90%, and LYM% ≤ 2.11%.

### Load Protocol

Because of the practical relevance of this study, daily routines should not be disturbed. Daily routines involved school, homework, free time activities and training. Thus, the overall load was multifactorial and covered psycho-emotional (school and free time activity) and physical (training) strains. The respective training sessions were either performed in small groups or individually. On day 1, training routine covered coordination (50%/33%) and endurance exercises (50%/67%). The group was divided in morning training only (*n* = 2), afternoon training only (*n* = 5) or morning and afternoon training (*n* = 7) on day 2. Morning training sessions focused on coordination (20%), power (40%), and endurance (40%). In the afternoon sessions, athletes either combined coordination (20%), power (40%), and endurance (40%) exercises, performed strength training only (100%) or combined strength training (75%) and power exercises (25%). On day 3, six athletes focused on strength exercises only (100%) whilst one combined strength training (70%) and power exercises (30%). The other seven athletes had a day without training. Please see Supplementary Table 1 for the detailed training protocol.

### Statistical Analyses

We used a generalized estimating equation (GEE) approach to evaluate changes in capillary immunological blood markers and inflammatory indexes from post-training to the next morning. GEE is suited for dealing with randomly missing data and repeated measurements (i.e., 3 days with two measurements per day). Furthermore, it produces robust parameter estimates and standard errors (Zeger and Liang, 1986; Burton et al., 1998). Blood markers were entered into GEE models as dependent variables. The time of day (dichotomous: post-training vs. morning) was used as independent variable for this statistical model. We used a Gaussian link function and within-subject dependencies were modeled as first-order autoregressive. To examine significant differences between time of day, we estimated pairwise contrasts of the marginal means. Changes in capillary immunological blood markers between morning measurements, were analyzed using GEE. Capillary immunological blood markers were *z*-transformed and entered as dependent variables. The day of measurement was entered as independent variable. We reported marginal means and 95% confidence intervals for all training days. We reported Bonferroni-Holm corrected *p*-values for post-training to the next morning and morning to morning changes.

GEE was also used to evaluate interrelation between subjective (3 Items) and objective measures (10 items). Therefore, only full datasets were included. First, all parameters were *z*-transformed. For our GEE models, the measures of sleep “sleep duration” and “sleep depth” were entered as independent variables. Post-training to morning change-scores of capillary immunological blood markers were entered separately as dependent variables. We reported standardized regression coefficients. According to, Fritz et al. (2012), *r* = 0.5; 0.3; 0.1 is referred to large, medium and small effects, respectively. Two-sided *p*-values were reported. The significance level was set to α = 5%. Data analyses was performed using SPSS (Version 22, IBM, United States) and R (R Foundation for Statistical Computing, Vienna, Austria).

## Results

Due to signs of upper respiratory tract infections for more than 48 h, one athlete (KS008) was excluded from data analyses. Another athlete (KS009) attended two measurements only and was therefore excluded by reason of missing data.

### Immune Regulation

**Figure 2:**
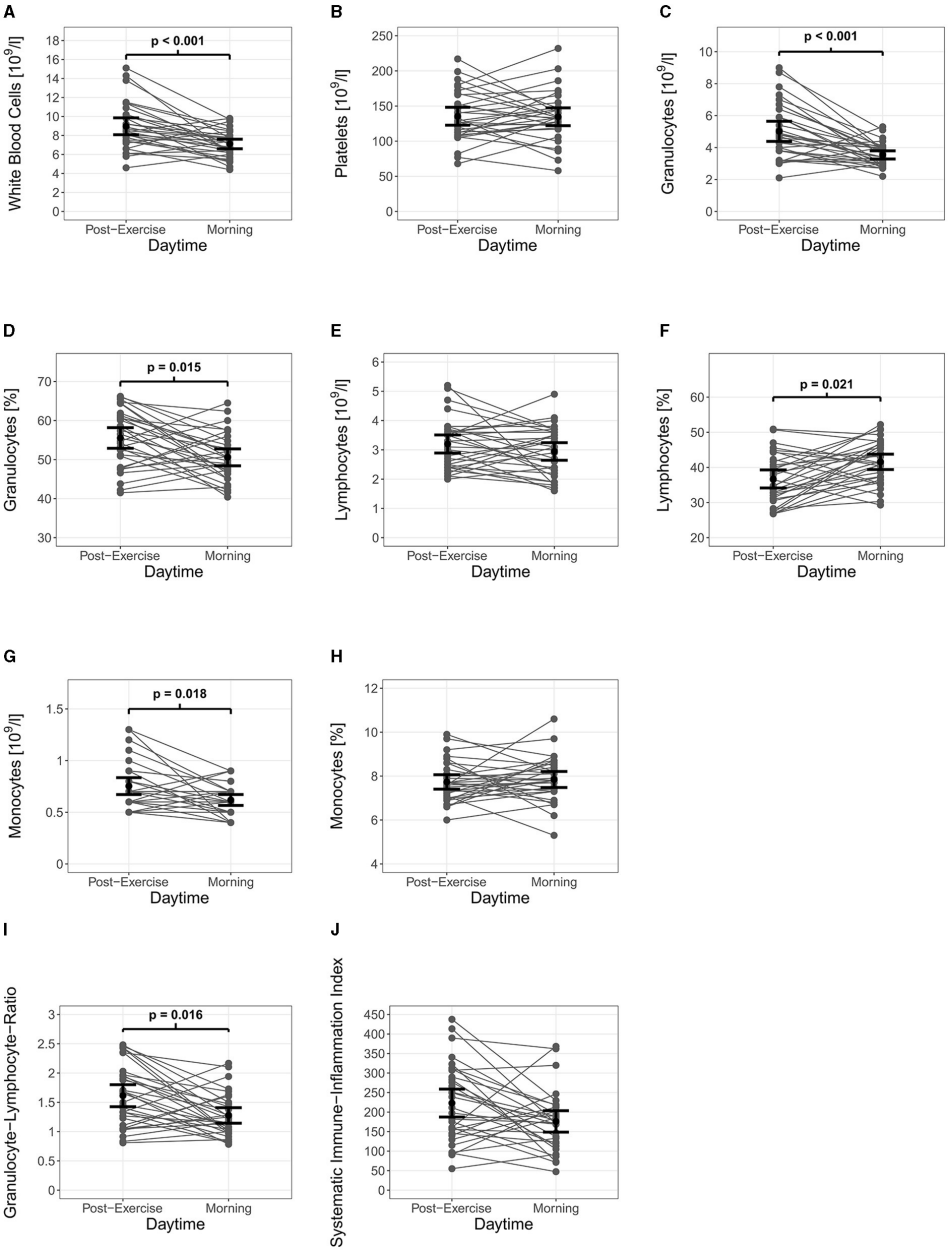
Immune regulation of capillary immunological blood markers and inflammatory indexes with 95% CI. White blood cells **(A)**, granulocytes **(C)**, granulocytes % **(D)**, monocytes **(G)** and granulocyte-lymphocyte-ratio **(I)** decreased significantly from post-training to the next morning. Lymphocytes % **(F)** showed significant decreases, while platelets **(B)**, lymphocytes **(E)**, monocytes % **(H)**, and systemic inflammation index **(J)** showed no significant changes.

Pairwise comparisons from post-training sessions to the next morning (32 datasets with 64 observations) revealed significant differences in capillary immunological blood markers. As displayed in Table 1, WBC (*p* < 0.001), GRAN (*p* < 0.001), GRAN% (*p* = 0.015), MID (*p* = 0.018), and GLR (*p* = 0.016) decreased significantly from post-training to the next morning. In contrast, LYM% showed a significant increase (*p* = 0.021) overnight. Differences in capillary immunological blood markers exceeded Intra-Assay and Inter-Assay Coefficients of Variability. Furthermore, the granulocyte-lymphocyte-ratio significantly decreased from the last training session of the day to the next morning (*p* = 0.016). For systemic inflammation, the analysis revealed a trend for a decrease from last training session of the day to the next morning (*p* = 0.072). Figure 2 presents individual single night changes in capillary immunological blood markers, granulocyte-lymphocyte-ratio and systemic inflammatory index from post-training sessions to the next morning.

**Table 1:** Marginal means and confidence intervals (95% CI) resulting from generalized estimating equation models for capillary immunological blood markers.

		**Post-training**			**Morning**			***p*_**raw**_**	***p*_**adj**_**
		**Mean**	**95% CI**		**Mean**	**95% CI**			
White blood cells	[10^9^/l]	9.12	8.15	10.08	7.33	6.62	8.04	<0.001	** <0.001**
Platelets	[10^9^/l]	136.11	119.3	152.93	136.51	119.2	153.81	0.962	0.999
Granulocytes	[10^9^/l]	5.08	4.41	5.74	3.65	3.28	4.03	<0.001	** <0.001**
Granulocytes	[%]	55.58	53.22	57.94	50.76	47.52	53.99	0.003	**0.015**
Lymphocytes	[10^9^/l]	3.26	2.96	3.56	3.01	2.59	3.43	0.211	0.633
Lymphocytes	[%]	36.69	34.35	39.02	41.38	38.07	44.68	0.003	**0.021**
Monocytes	[10^9^/l]	0.76	0.67	0.85	0.63	0.56	0.7	0.003	**0.018**
Monocytes	[%]	7.72	7.36	8.08	7.83	7.37	8.29	0.546	0.999
Granulocyte-lymphocyte-ratio	–	1.61	1.44	1.79	1.29	1.09	1.49	0.002	**0.016**
Systemic inflammation index	–	223.72	193.62	253.83	179.28	141.05	217.5	0.018	0.072

*Bold values indicate significant differences (p < 0.05)*.

**Figure 3:**
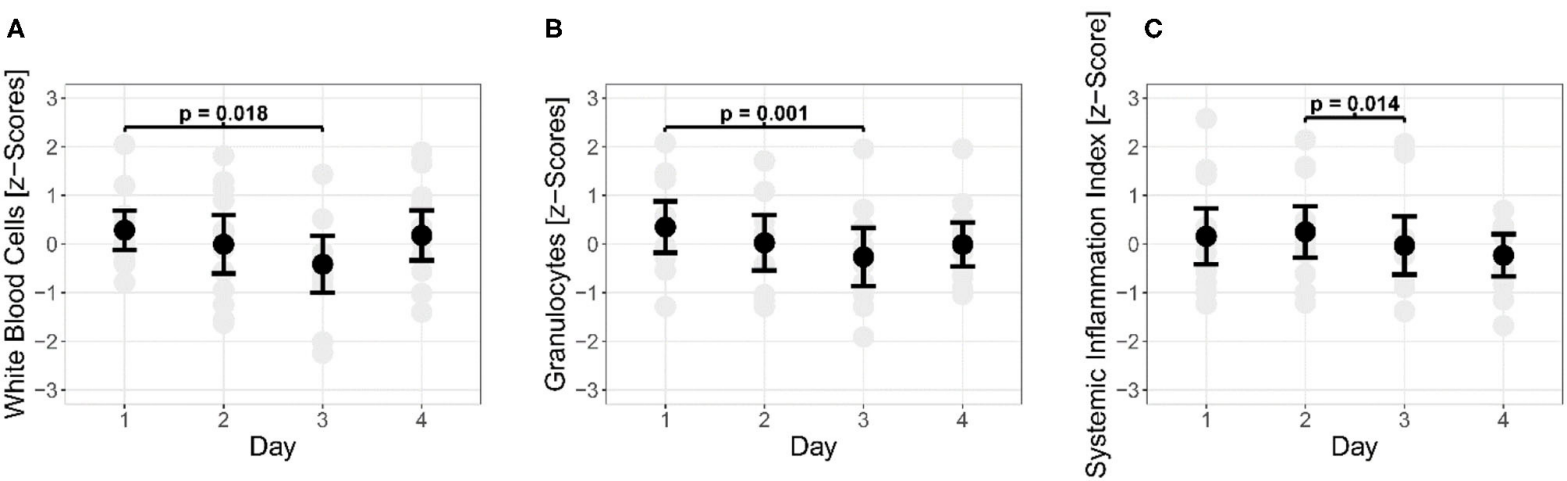
Changes during morning measurements of white blood cells **(A)** and granulocytes **(B)** and the Systemic Inflammation Index **(C)** over a 4-day (three nights) observational period.

A total of 12 athletes (52 observations) were included for morning to morning analyses. There were significant differences in capillary immunological blood markers throughout the days. From morning 1 to morning 3, WBC (*p* =0.018) and GRAN (*p* = 0.001) decreased significantly. In addition, SII decreased significantly (*p* = 0.014) from morning 2 to morning 3 (see Figure 3). The morning of day 4 showed no significant difference to the first measurement (*p* > 0.05).

### Interrelation of Sleep Outcomes With Overnight Immune Regulation

**Figure 4:**
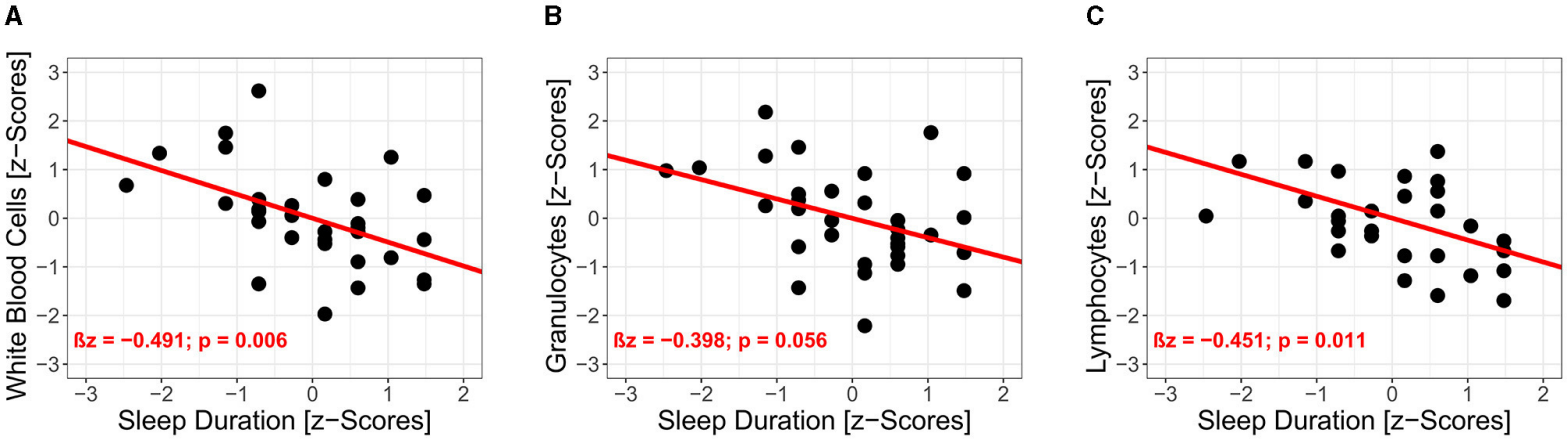
Interrelations of post-training to morning immune regulation in capillary blood markers and sleep duration. Results of the univariate regression analyses are illustrated for white blood cells **(A)**, granulocytes **(B)** and lymphocytes **(C)**.

Results of the univariate regression analysis (32 datasets with 64 observations) are illustrated in Table 2. There were significant associations between sleep duration and capillary immunological blood markers (see Figure 4). Sleep duration was negatively associated with WBC (standardized regression coefficient β*z* = −0.491, 95% CI: −0.772 to −0.210, *p* = 0.006) and LYM (β*z* = −0.451, 95% CI: −0.724 to −0.178, *p* = 0.011). Further, GRAN (β*z* = −0.399, 95% CI: −0.688 to −0.109, *p* = 0.056) and MID (β*z* = −0.468, 95% CI: −0.893 to −0.043, *p* = 0.217) were negatively associated with sleep duration but did not reach the level of significance. Sleep depth showed no significant interrelations with capillary immunological blood markers.

**Table 2:** Results of the univariate regression analysis using generalized estimating equations for capillary immunological blood markers and sleep duration.

**Change-scores**			**Sleep duration**			
		***βz***	**95% CI**		***p*raw**	***p*adj**
White blood cells	*z*-score	−0.491	–0.772	–0.210	<0.001	**0.006**
Platelets	*z*-score	0.017	–0.321	0.356	0.920	0.920
Granulocytes	*z*-score	−0.399	–0.688	–0.109	0.007	0.056
Granulocytes %	*z*-score	0.054	–0.224	0.332	0.710	0.999
Lymphocytes	*z*-score	−0.451	–0.724	–0.178	0.001	**0.011**
Lymphocytes %	*z*-score	–0.065	–0.351	0.222	0.660	0.999
Monocytes	*z*-score	−0.468	–0.893	–0.043	0.031	0.217
Monocytes %	*z*-score	0.160	**–**0.170	0.490	0.340	0.999
Granulocyte-lymphocyte-ratio	*z*-score	–0.082	–0.393	0.229	0.610	0.999
Systemic inflammation index	*z*-score	–0.043	–0.292	0.206	0.730	0.999

*Bold values indicate significant differences (p < 0.05)*.

## Discussion

The aim of this observational study was to examine the associations of overnight immunological regulation and subjective measures of sleep (e.g., sleep duration and sleep depth) in male and female adolescent track and field athletes. Our data showed significant decreases (WBC, GRAN, MID, GRAN%, GLR, a trend for SII) and increases (LYM%) from the last training session of the day until the next morning in selected capillary immunological blood markers over the 4 day (three nights) study period. A comparison between morning measurements revealed significant changes in WBC, GRAN, and SII, indicating a not systematic immune regulation from morning to morning. In addition, we found that sleep duration was negatively associated with the overnight immune regulation.

Our data showed that WBC, GRAN, GRAN%, MID, and GLR decreased significantly, whereas LYM% significantly increased from post-training to the next morning. There is an inverse immune cell kinetic compared to training-induced immunological stress responses (Puta et al., 2018). Decreases in WBC, GRAN and MID are likely caused through a migration in lymphatic organs (Gabriel and Kindermann, 1997; Cermakian et al., 2013; Scheiermann et al., 2013). In addition, the downregulation of SNS and HPA at night (Lange et al., 2010; Irwin and Opp, 2017) decreases the migration from marginal pool into the blood circulation. These results are in line with the literature. Accordingly, Born et al. (1997) investigated the influence of sleep on immune cell kinetics in healthy men within a 51 h sleep wake cycle and demonstrated, that immune cells follow a circadian rhythmicity over a 24 h sleep-wake cycle. Thus, WBC, NEUT, LYM, and MID decrease from post-training to morning. Thereby, WBC, NEUT, and MID show their nadir around 8:00 a.m. while LYM increases during the night but decreases again until 11:00 a.m. Non-significant decrease in LYM at 8:00 a.m. seems to depend on normal circadian rhythm. This implicates that circadian rhythm is not disturbed by training-induced immunological stress responses. Although, systemic inflammation indexes (GLR, SII) increases after intensive exercise (Joisten et al., 2019; Schlagheck et al., 2020; Wahl et al., 2020), overnight immune regulation seems so have a systemic effect. In support, a significant decrease overnight in GLR and a trend for decrease in SII (*p* = 0.056) were reported by Joisten et al. (2019), who investigated male sport students in a randomized controlled trial. Further, the authors reported significant increases in the neutrophil-lymphocyte-ratio and SII after a single exercise session with 300 countermovement jumps. Immunological stress responses were balanced throughout 24 h post-exercise. Differences in the exercise procedure may explain the variance in post-training measurements since heavy eccentric exercise causes more local tissue damage and therefore provoke a stronger immune response (Joisten et al., 2019). Furthermore, our observational study included psycho-emotional strains which occur also in advance of the training. To our knowledge this is the first study investigating the overnight immune regulation in adolescent athletes. Previous studies primarily focus on changes in immunological blood markers over several nights and their association to sleep deprivation (Dinges et al., 1994; Kerkhofs et al., 2007; Boudjeltia et al., 2008; Faraut et al., 2011; Pérez de Heredia et al., 2014). Therefore, a comparison to these studies does not seem to be appropriate. With reference to our results, it is interesting that participants showed interindividual differences in immune regulation from post-training to the next morning (see Supplementary Figure 1). For instance, KS002, KS014, and KS020 showed decreases in WBC in three comparisons, whereas KS001 increased twice from post-training to the next morning. This indicates that overnight immune regulation is highly individual. Nonetheless, interindividual differences may be referred to different training frequency, volume and intensity. Therefore, future research should aim to evaluate interindividual differences in immune regulation throughout standardized exercise protocols.

Results showed significant changes in acute (WBC, GRAN) and systemic inflammation markers (SII) from post-training to the next morning throughout the 3-night period. Morning measurements of capillary immunological blood markers did not decrease after 1 day of training, whereas a significant decrease was found after 2 days of training compared to the first morning. Those changes may reflect the training protocol, as different training methods were applied which differed in terms of exercise intensity and frequency (Table 1). On the other hand, differences between morning values on day 1 and 3 are likely due to an increased migration to damaged tissue or lymphatic organs in the course of local inflammation. Hence, adolescent athletes were more vulnerable to immunological challenges on morning of day 3 compared to the morning of day 1. Both, a day off (*n* = 6) or an additional strength training session (*n* = 7) increased WBC and GRAN in a meaningful magnitude from the morning of day 3 to the morning of day 4. Of note, there was no significant difference in WBC and GRAN between the morning of day 1 and the morning of day 4. SII decreased significantly from the morning of day 2 to the morning of day 3, reflecting decreased systemic low level inflammatory immune regulation. Interestingly, the results from the morning of day 3 showed no significant difference to the results obtained from the morning of day 1 and 4. Thus, our data indicate, that a repetitive application of track and field training on consecutive days is associated with decreases in WBC and GRAN. In our study the associations with systemic inflammatory markers (GLR, SII) were not systemically observed. This means that either recovery (e.g., sleep) was sufficient in terms of low level inflammatory immune regulation or that the systemic inflammatory markers (i.e., GLR, SII) are not sensitive enough in adolescent track and field athletes. However, repetitive, intensive training sessions on consecutive days should be counterbalanced by recovery days, including sufficient sleep. In order to determine immunological recovery duration, training-specific mechanical (e.g., plyometric vs. agility), metabolic and hormonal impacts on immunity have to be considered.

Changes in capillary immunological blood markers showed significant interrelations to sleep duration (WBC, LYM) and a trend in GRAN. Accordingly, extended sleep duration is associated with decreases in WBC, LYM, showing evidence for the link between sleep and the immune system. This may be due to sleep-induced downregulation of HPA axis and SNS, resulting in lower catecholamine release and lower pro-inflammatory cytokine secretion (Born et al., 1997; Lange et al., 2010; Del Gallo et al., 2014; Ibarra-Coronado et al., 2015; Irwin and Opp, 2017). In fact, these findings confirm our hypothesis. Moreover, our results are in line with the literature. Thus, Pérez de Heredia et al. (2014) demonstrated that sleep duration is negatively associated with WBC, NEUT, and MID in adolescents. In this context, it should be mentioned that the results of Pérez de Heredia et al. (2014) showed significant but weak associations based on estimated mean sleep duration, whereas our results considered sleep duration for each night separately. A negative association of sleep duration immunological blood markers was previously reported by studies, investigating sleep restriction and sleep loss (Dinges et al., 1994; Boudjeltia et al., 2008; Faraut et al., 2011). In contrast, we investigated the acute effects of regular sleep patterns in adolescent track and field athletes. Although, our findings are in line with Born et al. (1997), we did not apply experimental sleep restriction or partial sleep deprivation. However, we assume, that sleep deprivation and/or sleep loss would impair immune regulation following track and field training in adolescent athletes. Further, sleep depth showed no significant interrelations with immunological blood markers. Particularly sleep depth was expected to be associated with changes in capillary immunological blood markers. Sleep depth is referred to non-rapid eye movement (NREM) phases of sleep (Irwin and Opp, 2017). Because of its link to SNS activity (Somers et al., 1993), NREM is likely to be associated with a reduction of WBC and NEUT (Irwin and Opp, 2017). Although NREM and sleep depth represent the same aspect of sleep, differences in detailed assessment of sleep architecture and single item query have to be considered. Therefore, and supported by the literature (Nieman and Wentz, 2019), the assessment of sleep duration and sleep depth should be implemented in the adolescent athlete's training routine. However, due to the essential differences between subjective and objective (e.g., polysomnography) sleep measures (Baker et al., 1999; Lauderdale et al., 2008), our results only account for the assessment of subjective sleep measures.

This study was carried out as an observational study with an multifactorial load (psycho-emotional, physical), representing the strains of the whole day. Thus, the explicit effect of training on sleep duration and immunological regulation cannot be derived from our findings. Of note, stress dimension of the Acute Recovery and Stress Scale (Kellmann et al., 2016) showed no significant difference (*p* > 0.05) between post-training measurements (see Supplementary Figure 2). Track and field training was individualized and not standardized for content of training, frequency, volume and intensity. From a practitioner's perspective, we suggest to monitor at least overall training intensity, when evaluating immune regulation in adolescent athletes. As training sessions were performed in groups, with different frequencies, the differences in the individual's training routine are an important factor when interpreting our results. Although, post-training assessment time varied within the group, they were performed 30–45 min after the last training session. With respect to ongoing shifts in WBC subsets after exercise (McCarthy and Dale, 1988; Gabriel and Kindermann, 1997), immunological responses to track and field training were not at its peak. However, the relation to peak immunological responses was not subject to this study. Morning capillary blood assessment was carried out between 7:00 and 10:00 a.m. Therefore, each participant was measured on a different time point. This may result in interindividual differences in capillary immunological blood markers. More precise, due to circadian rhythmicity (Born et al., 1997), NEUT increases and LYM decreases from 7:00 to 10:00 a.m. Results from sleep assessment obtained from questionnaires differ significantly from those obtained from the objective assessment (Baker et al., 1999; Lauderdale et al., 2008). With respect to accuracy, questionnaires are limited to rating biases and retrospective estimation, which is why an additional objective measurement should be implemented for future studies. Since a permanent training monitoring cannot be applied under laboratory conditions, actigraphy would be a possible, but also expansive option for objective sleep assessment. Because they may not be affordable for adolescent athletes, subjective measures of sleep are a justified and acceptable approach but findings have to be validated through objective sleep assessment (e.g., actigraphy).

As training intensity and volume effect the magnitude of training-induced immunological stress responses, our findings only apply immune regulation in adolescent track and field athletes. Sample size could not be calculated prior to the study, due to missing data on immunological regulation following track and field training and its association to undisturbed sleep behavior. Further, our study was addressed to a specific group of young athletes with a similar training protocol in terms of training intensity and volume. Therefore, sample size was limited to the size of the training group. Statistical power depends on sample size and number of repeated measures. GEE does not need a particular distribution of outcome measures. Although, it is suited to deal with small sample sizes (*n* = 8) and repeated measures (*n* = 6), while at the same time achieve around 80% power (Ma et al., 2012), the results of our field study have to be validated through prospective studies with a larger sample size.

We recommend coaches to estimate the impact of track and field training on the immune system when planning their training schedules. Interindividual differences have to be taken into account. Sufficient recovery time is necessary to develop athletic and sport-specific performance while at the same time, promoting health and to avoid injuries. Sleep appears to be an important factor for immune regulation in adolescent track and field athletes. Therefore, sleep assessment protocols should be established for adolescent athletes. Thereby, practicability and costs have to be considered.

Future research should focus on optimizing sleep assessment to meet the demands for immune regulation in adolescent athletes. However, sleep duration is an easy parameter to assess, which shows significant relations to immune regulation in adolescent athletes. Further, relevant measures of sleep have to be identified in order to estimate overnight immune regulation. Thereby, sleep assessment should be applied for a minimum of three consecutive nights. The circadian rhythm is a process that is independent from homeostatic measures (e.g., sleep duration, sleep depth) and is well-known for its influence on immune cell kinetics (Besedovsky et al., 2019). This includes power-naps, which support immune regulation following sleep restriction up to 2 h a single night (Faraut et al., 2011).

Sleep restriction is an immunological challenge that provokes immune cell activation (Del Gallo et al., 2014; Ibarra-Coronado et al., 2015; Irwin and Opp, 2017; Prather, 2019). Therefore, short-term partial sleep restriction during times of low training intensity and volume may promote immunological adaptations. A potential immunological benefit of short-term partial sleep restriction is not investigated and should be subject to future studies.

## Conclusion

Our findings indicate that overnight immune regulation is associated with subjective measures of sleep duration in experienced adolescent track and field athletes. Therefore, overnight sleep-dependent immune regulation may have important implications for the recovery and performance on the day after. Trainers and practitioners should pay attention to sleep behavior in adolescent athletes, especially for young competitive athletes. Particularly, overnight sleep duration should be monitored in order to facilitate sufficient immunological regulation ahead on the next training session. Future research should focus on determining dose-response relations of measures of sleep and immune regulation in response to multifactorial stress in athletes. In this context, studies should aim to examine the acute effects of a single night but also additive effects over consecutive days of training in adolescent athletes.

## Data Availability Statement

The original contributions presented in the study are included in the article/Supplementary Material, further inquiries can be directed to the corresponding authors.

## Ethics Statement

The studies involving human participants were reviewed and approved by University Research Ethics Committee of the Friedrich-Schiller-University Jena (458510/15). Written informed consent to participate in this study was provided by the participants' legal guardian/next of kin.

## Author Contributions

TS, UG, and CP: designed the experiment. RM and MH: gathered data. TS, PB, BG, and CP: conducted data analysis. TS, MK, WB, HG, and CP: wrote the manuscript. All authors discussed the results and its implications, commented and edited the manuscript at all stages, and approved the final version.

## Funding

This study is part of the research project Resistance Training in Youth Athletes that was funded by the German Federal Institute of Sport Science (ZMVI4-081901/20-23).

## Conflict of Interest

The authors declare that the research was conducted in the absence of any commercial or financial relationships that could be construed as a potential conflict of interest.

## Publisher's Note

All claims expressed in this article are solely those of the authors and do not necessarily represent those of their affiliated organizations, or those of the publisher, the editors and the reviewers. Any product that may be evaluated in this article, or claim that may be made by its manufacturer, is not guaranteed or endorsed by the publisher.

## Supplementary Material

Supplemental Table 1 Track and field athlete's training routine for each participant identification number (ID).

Supplemental Figure 1 Interindividual immune regulation.

Supplemental Figure 2 Post-training measurements of the Acute Recovery and Stress Scale (ARSS) showed no significant day-to-day differences for the stress dimension (1–2: *p* = 0.477; 1–3: *p* = 0.477; 2–3: *p* = 0.982).
