# Supplementary material for: Overnight Immune Regulation and Subjective Measures of Sleep: A Three Night Observational Study in Adolescent Track and Field Athletes
Source: Front Sports Act Living. 2021 Sep 28;3:689805. doi: 10.3389/fspor.2021.689805 (PMC8506008; doi:10.3389/fspor.2021.689805)
Supplement: Supplemental Table 1 — Track and field athlete's training routine for each participant identification number (ID). [file Table_1.DOCX]

|  | **Day 1** | | **Day 2** | | **Day 3** | |
| --- | --- | --- | --- | --- | --- | --- |
|  | **ID** | **No training** | **ID** | **Training (TR2-1)** | **ID** | **Training (TR3)** |
| **Morning** |  |  | KS009 KS010 | **coordination (15 min)**  **speed (30 min)** 3x30 m (3 min rest)  5x20 m, (2 min rest) **anaerobic endurance (30 min)**  3x90 m (4 min rest)  3x120 m (6 min rest) | KS001  KS003  KS005  KS009  KS010 KS018 | **heavy resistance strength training (hypertrophy, 3 sets, 8 repetitions, 60 min)** Benchpress Rowing  Dead lifts  Legpress (eccentric)  Single-leg squats  Hip flexion |
|  |  |  | **KS007 KS008 KS012 KS014** | **coordination (hurdles,  15 min)**  **speed (hurdles, 40 min)**  3x30 m (3 min rest)  5x 2^nd^ hurdle 3-P-Start (2 min rest)  **anaerobic endurance (30 min)**  3x90 m (4 min rest)  3x120 m (6 min rest) | KS016 | **heavy resistance strength training (hypertrophy, 3 sets, 8 repetitions, 40 min)** Benchpress  Dead lifts  Single-leg squats  Hip flexion  **horizontal jumping (20 min)** 3x30 m single-leg alternate  3x30 m single-leg right  3x30 m single-leg left |
|  |  |  | **KS002 KS004 KS020** | **coordination (15 min)**  **speed (30 min)** 3x30 m(3 min rest)  5x20 m (2 min rest) **anaerobic endurance (40 min)**  5x120 m 90% (6 min rest) | KS002  KS004 KS007  KS008  KS012 KS014  KS020 | **Day off** |
|  | **ID** | **Training (TR1)** | **ID** | **Training (TR2-2)** | **ID** | **Training** |
| **Afternoon** | KS001 KS005 KS007 KS008 KS009 KS010 KS012 KS014 KS016 | **dynamic core stability (20 min)**  **aerobic extensive endurance (20 min)**  10x120 m (walking rest) | KS001 | **coordination (15 min)**  **speed (30 min)** 3x30 m (3 min rest)  5x20 m (2 min rest) **anaerobic endurance (30 min)**  3x90 m (4 min rest)  3x120 m (6 min rest) |  |  |
|  | KS002 KS003 KS004 KS018 KS020 | **dynamic core stability (20 min)**  **aerobic extensive endurance (40 min)**  10x250 m (walking rest) | KS005 KS016 | **coordination (hurdles,  15 min)**  **speed (hurdles, 40 min)**  3x30 m (3 min rest)  5x 2^nd^ hurdle 3-P-Start (2 min rest)  **anaerobic endurance  (30 min)**  3x90 m (4 min rest)  3x120 m (6 min rest) |  |  |
|  |  |  | **KS002 KS004 KS007 KS008 KS012 KS020** | **heavy resistance strength training (hypertrophy, 3x8 repetitions, 60 min)** Benchpress Rowing  Dead lifts  Legpress (eccentric)  Single-leg squats  Hip flexion |  |  |
|  |  |  | **KS003 KS018** | **coordination (15 min)**  **speed (30 min)** 3x30 m (3 min rest)  5x20 m (2 min rest) **anaerobic endurance  (40 min)**  5x120 m 90% (6 min rest) |  |  |
|  |  |  | **KS014** | **heavy resistance strength training (hypertrophy,3x8 repetitions, 60 min)** Benchpress Rowing  Dead lifts  Legpress (eccentric)  Single-leg squats  Hip flexion **horizontal jumping (20 min)** 3x30 m single-leg alternate  3x30 m single-leg right  3x30 m single-leg left |  |  |
