# Supplementary figures and images for: Overnight Immune Regulation and Subjective Measures of Sleep: A Three Night Observational Study in Adolescent Track and Field Athletes
Source: Front Sports Act Living. 2021 Sep 28;3:689805. doi: 10.3389/fspor.2021.689805 (PMC8506008; doi:10.3389/fspor.2021.689805)

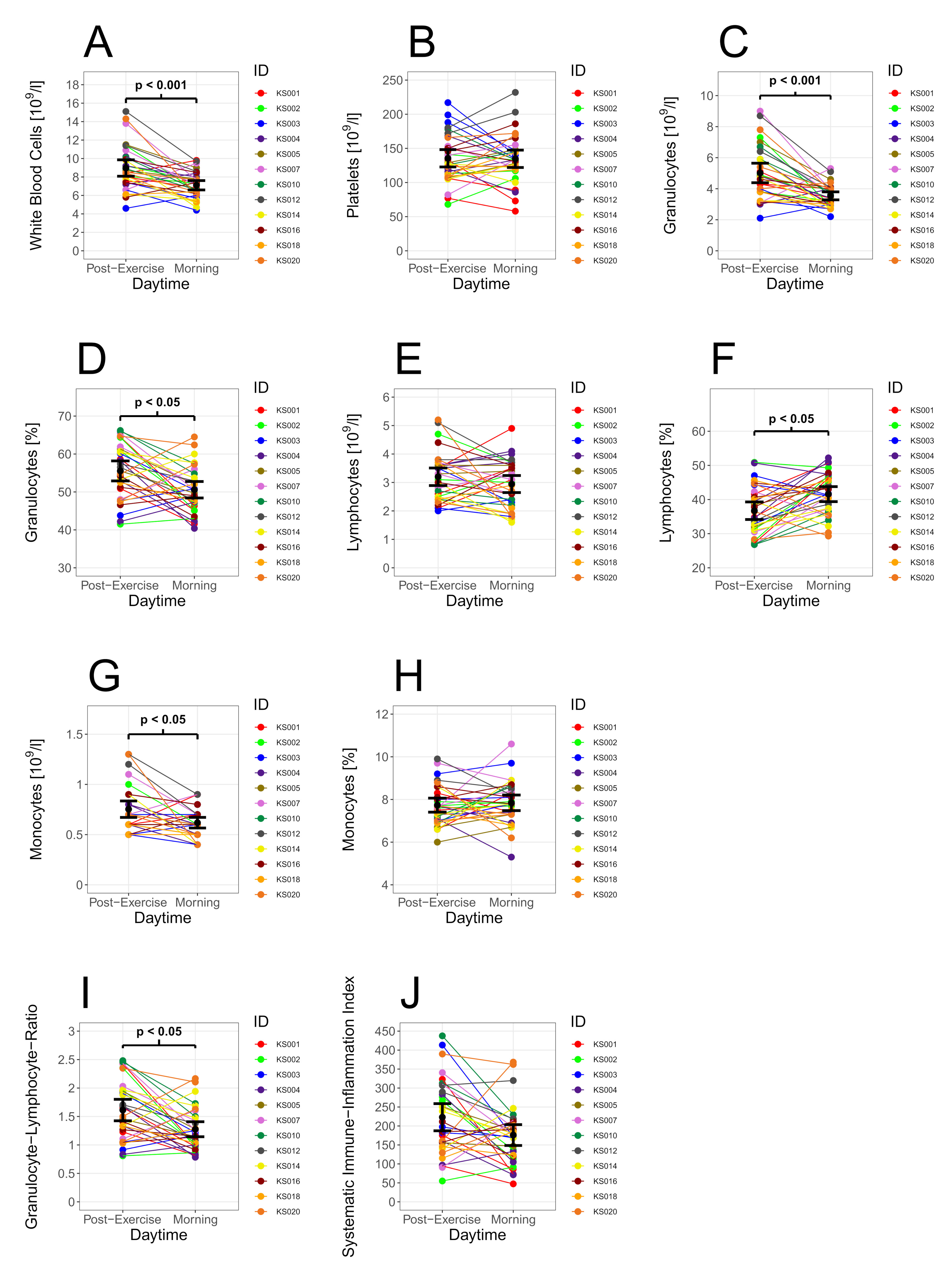

Supplement: Supplemental Figure 1 — Interindividual immune regulation. [file Image_1.JPEG]

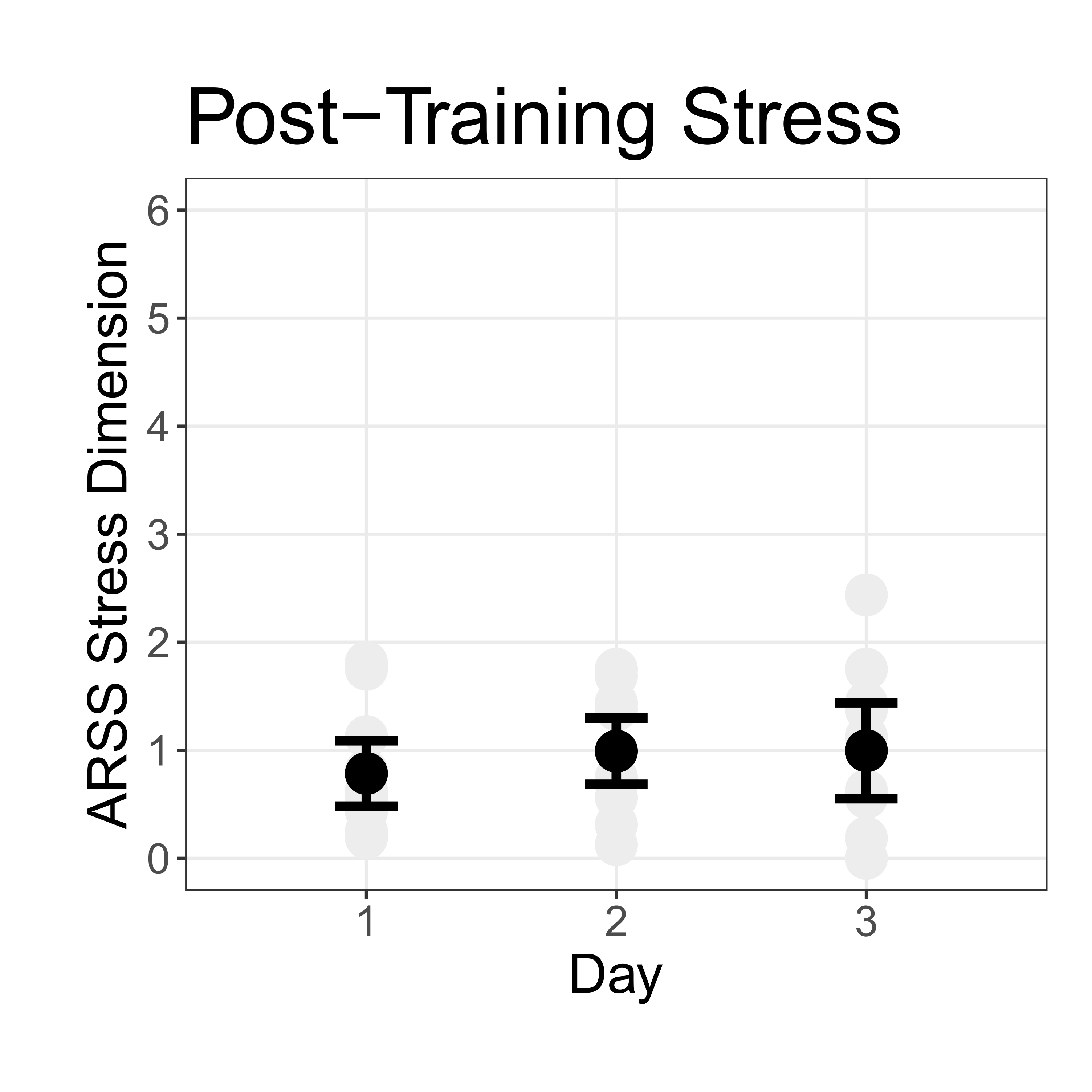

Supplement: Supplemental Figure 2 — Post-training measurements of the Acute Recovery and Stress Scale (ARSS) showed no significant day-to-day differences for the stress dimension (1–2: p = 0.477; 1–3: p = 0.477; 2–3: p = 0.982). [file Image_2.JPEG]
